# Neutrophil CD64 index: a novel biomarker for risk stratification in acute pancreatitis

**DOI:** 10.3389/fimmu.2025.1526122

**Published:** 2025-04-16

**Authors:** Min Shao, Ling Wu, Xiangping Huang, Qianhui Ouyang, Ya Peng, Sixiang Liu, Xu Xu, Qi Yi, Yi Liu, Guoguang Li, Ding Ning, Jia Wang, Chaochao Tan, Ying Huang

**Affiliations:** ^1^ Department of Clinical Laboratory, Hunan Provincial People’s Hospital (The First Affiliated Hospital of Hunan Normal University), Changsha, China; ^2^ Gastroenterology department, Hunan Provincial People’s Hospital (The First Affiliated Hospital of Hunan Normal University), Changsha, China; ^3^ Department of Emergency, The First Affiliated Hospital of Hunan Normal University (Hunan Provincial People’s Hospital), Changsha, China; ^4^ Hepatobiliary Surgery, Hunan Provincial People’s Hospital (The First Affiliated Hospital of Hunan Normal University), Changsha, China; ^5^ Department of Emergency Medicine, The Affiliated University of South China, Hengyang Medical School, University of South China, Changsha, China; ^6^ Hunan Provincial Key laboratory of Emergency and Critical Care Metabonomic, Hunan Provincial People’s Hospital (The First-Affiliated Hospital of Hunan Normal University), Changsha, China; ^7^ Tumor Immunity Research Center of Hunan Provincial Geriatric Institute, Hunan Provincial People’s Hospital (The First Affiliated Hospital of Hunan Normal University), Changsha, China

**Keywords:** acute pancreatitis, severe acute pancreatitis, neutrophil CD64 index, risk stratification, death prediction

## Abstract

**Objective:**

Effective early diagnosis and timely intervention in acute pancreatitis (AP) are essential for improving patient outcomes. This study aims to evaluate the clinical utility of the neutrophil CD64 index (nCD64) in stratifying patients with SAP and assessing mortality risk.

**Methods:**

A total of 302 AP patients were enrolled and divided into a training cohort (*n* = 226) and a validation cohort (*n* = 76). Venous blood samples were collected within 24 hours of admission, and the nCD64 index was measured via flow cytometry. Other clinical parameters, including C-reactive protein (CRP) and procalcitonin (PCT), were also recorded. Logistic regression and receiver operating characteristic (ROC) curve analyses were performed to assess the diagnostic value of the nCD64 index and its capacity to predict mortality risk.

**Results:**

ROC curve analysis identified a cutoff value of 1.45 for the nCD64 index. Patients with nCD64 > 1.45 had significantly higher risks of complications, including systemic inflammatory response syndrome (SIRS), acute respiratory distress syndrome (ARDS), multiple organ failure (MOF), and death. Over 65% of patients with acute pancreatitis (AP) can be effectively risk-stratified at a low cost, and it has been demonstrated that AP patients with an nCD64 value ≤ 1.45 have an extremely low mortality rate (no mortality in present training and validation cohort). Kaplan-Meier survival analysis revealed a significant survival difference between high-risk (nCD64 > 1.45) and low-risk groups (*p* < 0.001).

**Conclusion:**

The nCD64 index is an effective tool for early identification of SAP patients, allowing for the classification of over 65% of cases as low-risk for mortality.

## 1 Introduction

Acute pancreatitis (AP) is an inflammatory disorder of the pancreas, marked by complex etiology and acute clinical progression (1). It is typically characterized by severe abdominal pain, often accompanied by nausea and vomiting (2). In severe cases, complications such as intra-abdominal infections, pancreatic hemorrhage, and necrosis may occur. While most cases are mild, self-limiting, and resolve within one week, around 20% of patients progress to moderately severe or severe acute pancreatitis (MSAP/SAP), associated with complications such as bacterial infections, multi-organ failure, and pancreatic necrosis, with a mortality rate ranging from 20% to 40% (3, 4). Given the complexity of treatment, which often necessitates multidisciplinary management, early diagnosis and timely intervention are critical for optimizing patient outcomes. Early identification of high-risk patients facilitates the implementation of targeted therapeutic strategies and critical care support, thereby improving prognosis.

Effective risk stratification is crucial in identifying patients at higher risk of mortality early in the clinical course of AP (5). Timely and accurate assessment enables clinicians to allocate critical care resources appropriately, ensuring that patients with life-threatening complications receive immediate attention (6). By distinguishing between patients likely to experience a mild disease course and those at risk of rapid deterioration or death, healthcare providers can intervene earlier with more aggressive treatments, potentially reducing the mortality rate and preventing irreversible organ damage (7). Without efficient risk stratification, delayed or insufficient interventions may lead to worse outcomes, especially in cases of severe acute pancreatitis, where time-sensitive care is essential for survival (8, 9).

Despite the availability of various biomarkers for predicting the onset of SAP, limitations in their clinical application remain. Laboratory markers such as white blood cell count, C-reactive protein (CRP), and procalcitonin (PCT) offer some diagnostic value in SAP and are relatively easy to obtain (10–12). However, these markers lack sufficient sensitivity and specificity, limiting their ability to distinguish between mild and severe cases of pancreatitis. Additionally, scoring systems like the Acute Physiology and Chronic Health Evaluation II (APACHE II), Ranson score, and the Bedside Index for Severity in Acute Pancreatitis (BISAP) aim to improve the accuracy of SAP prediction (13). However, these systems require multiple clinical parameters, making them time-consuming and complex to calculate. In emergency settings, physicians need rapid and reliable results to guide treatment decisions, but the complexity of these scoring systems can delay timely interventions. Moreover, the applicability of these scoring systems across different regions and populations remains a concern. Therefore, despite the progress made, the current biomarkers and scoring systems still fall short in clinical practice, underscoring the need for more precise and rapid diagnostic tools for SAP.

In recent years, increasing attention has been given to immune monitoring in critically ill patients, particularly the role of neutrophil-related parameters. The neutrophil CD64 index (nCD64 index), a novel blood marker, has shown superior accuracy and speed in diagnosing sepsis and other critical conditions compared to traditional blood markers (14, 15). However, the clinical utility of the nCD64 index in predicting severe acute pancreatitis remains unclear (16–18). Further research is necessary to elucidate the potential of this marker in the context of AP.

This study aims to evaluate the clinical value of nCD64 in acute pancreatitis by measuring its levels upon hospital admission and assessing its potential as a reliable biomarker for predicting the severity of AP. Furthermore, the ability of nCD64 to contribute to effective risk stratification could significantly enhance the identification of high-mortality patients, allowing for the prioritization of critical interventions and better management of resources in life-threatening cases. The findings may provide valuable insights into early diagnostic strategies for SAP and equip clinicians with an effective tool for timely intervention.

## 2 Materials and methods

### 2.1 Study design

This study was conducted from May 2021 to December 2022 at Hunan Provincial People’s Hospital and Changsha Central Hospital, enrolling patients diagnosed with acute pancreatitis. Participants were prospectively recruited and simultaneously assigned to parallel training and validation cohorts at a 3:1 ratio, a methodology designed to minimize selection bias and ensure statistical validity.

**Figure 1:**
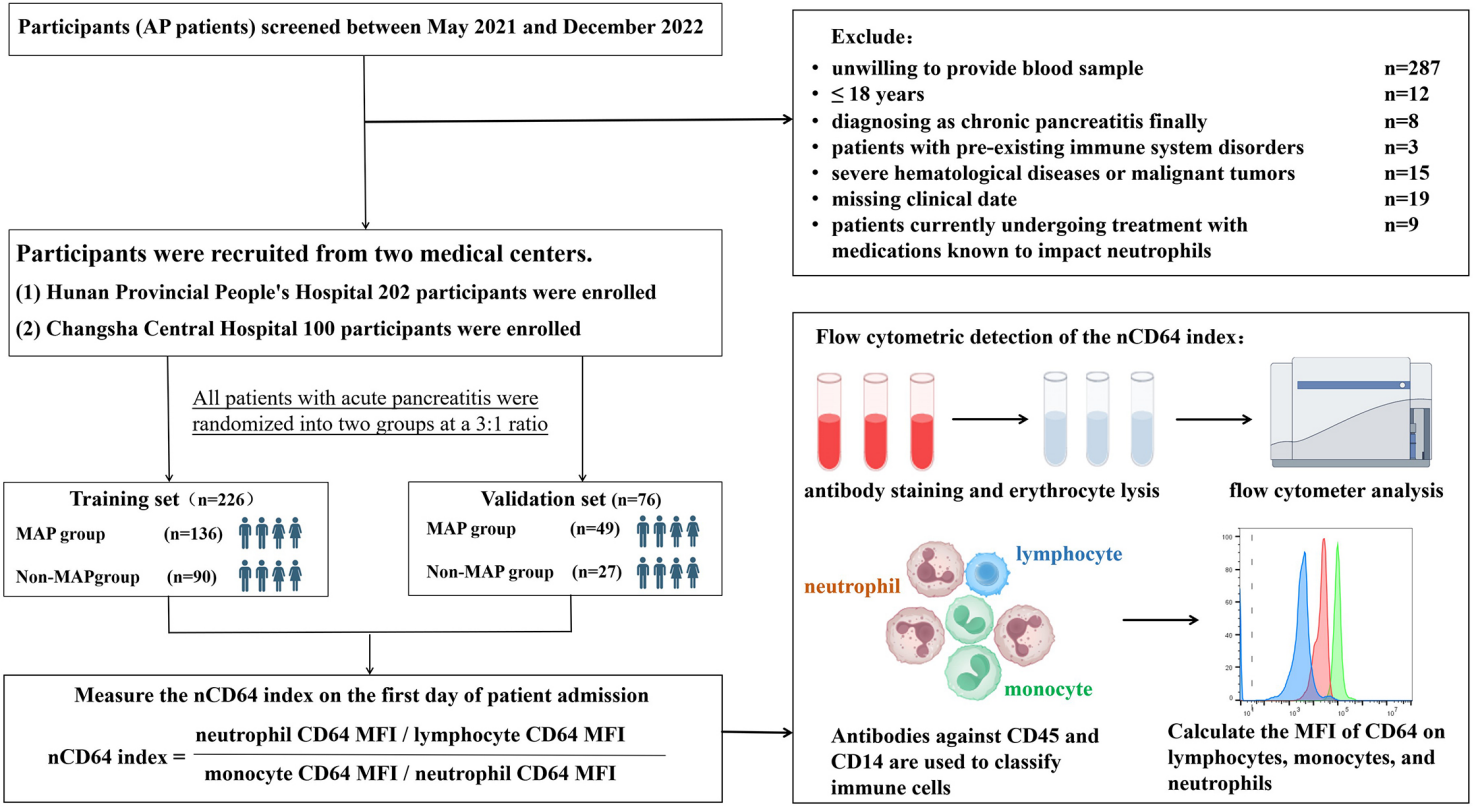
Study flowchart and experimental design. recruitment, exclusion, and randomization of acute pancreatitis (AP) patients into training and validation sets. The nCD64 index was measured using flow cytometry on the first day of admission, with immune cells classified by CD45 and CD14 antibodies. The nCD64 index was calculated as the ratio of neutrophil CD64 MFI to lymphocyte and monocyte CD64 MFI.

The sample size calculation was based on preliminary experiments and literature, using a one-tailed significance level (*α* = 0.05) and statistical power (1-β = 0.80). With an initial sensitivity of 0.857 observed in the experimental subset (*n* = 40) and a target sensitivity of 0.7, the required sample size for positive cases was 45. Given that positive cases represented ~20% of the population, the total estimated sample size was 226 for the training cohort. To align with the 3:1 allocation ratio and strengthen methodological rigor, an additional 76 participants were enrolled for validation, resulting in a final cohort of 302 participants: 226 in training and 76 in validation. This parallel cohort design ensured parameters derived from training were independently validated without overlap or adjustment. The detailed flow of participant screening is provided in **Figure 1**.

Within 24 hours of admission, venous blood samples were collected for nCD64 index quantification via flow cytometry, alongside clinical characteristics and standard laboratory biomarkers. The study adhered to the Declaration of Helsinki, with ethics approval from both institutions (Hunan Provincial People’s Hospital and Changsha Central Hospital, R201925). Written informed consent was obtained from all participants.

### 2.2 Study population

The diagnosis of AP was based on the diagnostic criteria, which required meeting at least two of the following three criteria: (1) persistent upper abdominal pain; (2) serum amylase and/or lipase concentration at least three times higher than the upper normal limit; (3) abdominal imaging findings consistent with acute pancreatitis (19–21).

Inclusion criteria: (1) meeting the diagnostic criteria for acute pancreatitis; (2) complete clinical data; (3) informed consent obtained; (4) age > 18 years.

Exclusion criteria: (1) patients with chronic or recurrent pancreatitis; (2) patients with a history of immune system diseases, including immunodeficiency diseases such as AIDS or systemic lupus erythematosus, rheumatoid arthritis, and systemic vasculitis; (3) patients with severe hematological disorders or malignancies; (4) patients currently undergoing treatment with drugs known to affect neutrophils, such as glucocorticoids or antithyroid drugs.

According to the revised 2012 Atlanta classification, AP patients were divided into three groups: MAP, characterized by no organ failure and no local or systemic complications; MSAP, characterized by transient (< 48 hours) organ failure and/or local complications or exacerbation of comorbidities; and SAP, characterized by persistent (> 48 hours) organ failure (4). The MSAP and SAP cases were categorized together as the MS-SAP group.

### 2.3 Reagents and instruments

Whole blood was collected using EDTA anticoagulant, and the nCD64 index was measured using flow cytometry (Myriad BriCyteE6, Mindray Medical, Shenzhen, China) within two hours of collection. Reagents for the nCD64 index assay included PerCP anti-human CD14, PerCP anti-human CD45, and PerCP anti-human CD64 (BioLegend, San Diego, CA, USA). The nCD64 detection procedure is summarized as follows: 50 μL of EDTA-anticoagulated blood sample was added to a test tube, along with 5 μL each of the CD14, CD45, and CD64 antibodies. The mixture was thoroughly combined and incubated in the dark for 15 min. Next, red blood cells were lysed, and the sample was processed for flow cytometry analysis. The mean fluorescence intensity (MFI) of CD64 expression on neutrophils, lymphocytes, and monocytes was determined.

**Figure 2:**
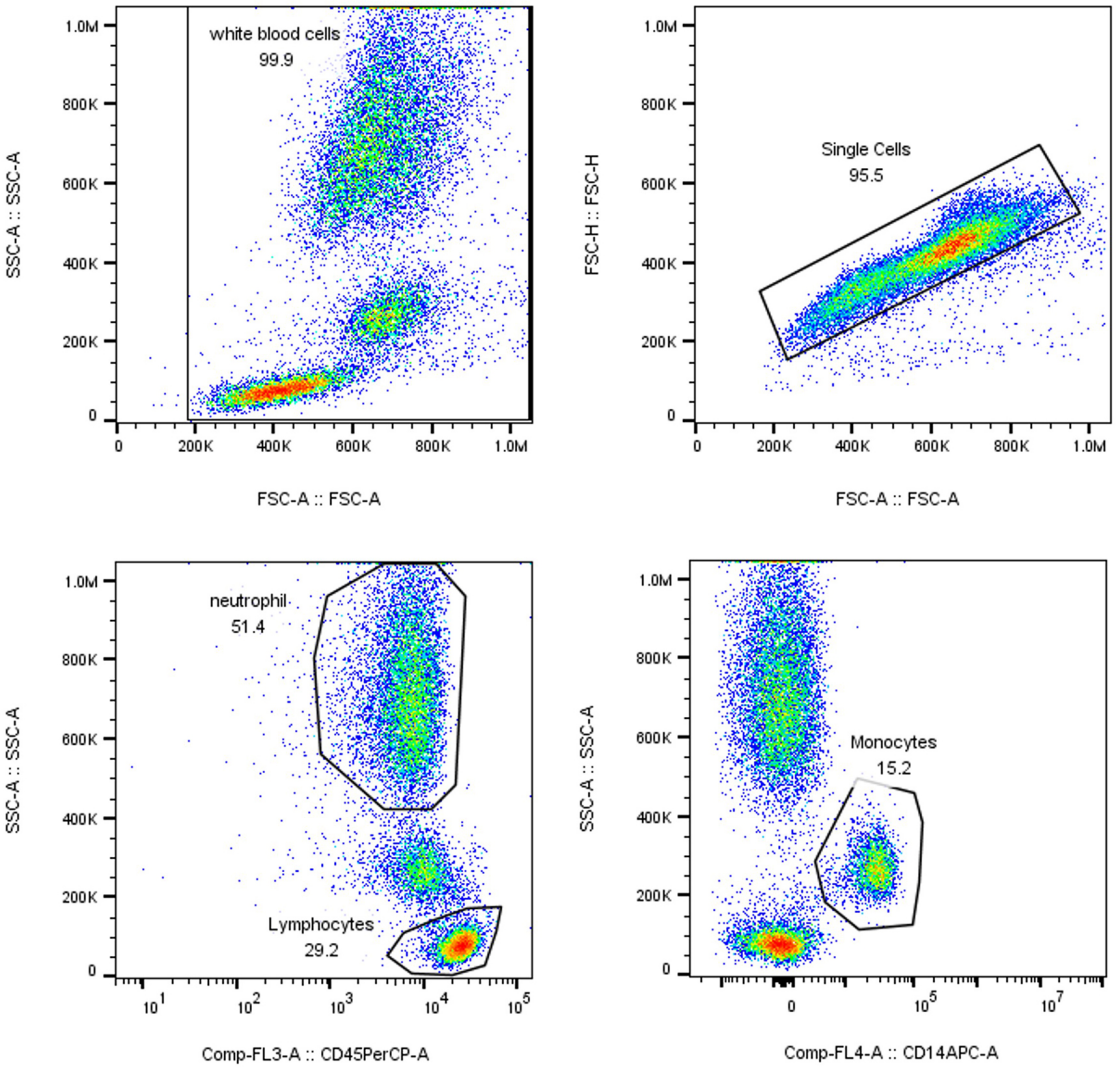
Gating Strategy. WBC count, immature granulocyte (IG) percentage, and neutrophil count (N) were analyzed using the XN Blood Analyzer Line and associated reagents.

nCD64 index = (neutrophil CD64 MFI/lymphocyte CD64 MFI)/(monocyte CD64 MFI/neutrophil CD64 MFI). The calculation of the nCD64 index accounted for intrinsic interindividual variability to ensure measurement stability and accuracy (since CD64 expression is highly stable on monocyte surfaces, it served as the positive control for the nCD64 index, whereas CD64 expression on lymphocytes is low, serving as the negative control). The gating strategy is illustrated in **Figure 2**.

### 2.4 Statistical analysis

Relevant clinical data, laboratory test results, and nCD64 index were analyzed using SPSS 23.0 and MedCalc software. The normality of continuous variables was assessed using SPSS. For normally distributed data, values were presented as mean ± standard deviation (x ± s). One-way analysis of variance (ANOVA) was used for comparisons among multiple groups, while the t-test was used for comparisons between two groups. For data with a skewed distribution that could be normalized, the transformed data were analyzed as normally distributed. If data could not be transformed, the median (interquartile range) (M [QL, QU]) was used, and comparisons between multiple groups and between groups were assessed using the Mann-Whitney U test. Comparisons of categorical data were conducted using the chi-square (χ²) test.

Additionally, we performed logistic regression analysis, Kaplan-Meier (K-M) curve analysis, and Spearman correlation analysis. Receiver operating characteristic (ROC) curve analysis was conducted using MedCalc software to assess the diagnostic efficacy of the nCD64 index and other indicators for severe acute pancreatitis. Subsequently, we compared the ROC curves obtained for multiple indicators in the same dataset to determine whether there were statistically significant differences in the predictive performance of each indicator. For the combined analysis of these indicators, we first conducted binary logistic regression analysis using SPSS. We then combined the two indicators, created a new composite index, and imported it into MedCalc for the aforementioned analysis.

## 3 Results

### 3.1 Baseline clinical characteristics of patients

As illustrated in **Table 1**, the baseline clinical characteristics of patients in both the MAP and MS-SAP groups. No significant differences were observed between the two groups regarding age or gender in either the training or validation cohorts (*P* > 0.05). However, there were significant differences in the length of hospital stay, with MS-SAP patients requiring longer hospitalization compared to MAP patients in both the training (*P* < 0.001) and validation (*P* = 0.081) cohorts.

**Table 1:** Comparison of clinical characteristics of patients.

Characteristics	Training set (*n* = 226)			Validation set (*n* = 76)			*P*-value^3^
	MAP (*n* = 136)	MS-SAP (*n* = 90)	*P*-value^1^	MAP (*n* = 49)	MS-SAP (*n* = 27)	*P*-value^2^	
Age [year, M (QL, QU)]	43.00(35.00, 52.75)	44.50 (35.75, 53.25)	0.582	45.00 (37.00, 53.00)	45.00 (37.00, 58.00)	0.896	0.325
Male [n (%)]	111.00 (81.60)	65.00 (72.20)	0.340	36.00 (73.50)	17.00 (63.00)	0.340	0.152
Days of hospitalization [Day, M (QL, QU)]	8.00 (5.00, 10.00)	15.00 (10.00, 21.00)	<0.001	7.00 (5.00, 9.50)	12.00 (6.00, 21.00)	<0.001	0.081
nCD64 index [M (QL, QU)]	1.16 (0.98, 1.32)	2.14 (1.62, 2.45)	<0.001	1.03 (0.93, 1.26)	2.01 (1.65, 2.42)	<0.001	0.327
CRP [mg/L, M (QL, QU)]	64.89 (5.86, 143.50)	123.50 (30.06, 204.27)	<0.001	11.10 (0.05, 118.62)	133.00 (29.61, 158.84)	0.001	0.052
WBC [×109/L, M (QL, QU)]	10.62 (7.63, 13.25)	11.22 (8.45, 15.52)	0.088	9.71 (7.22, 13.9)	9.87 (7.62, 16.78)	0.416	0.197
N [×109/L, M (QL, QU)]	8.21 (5.88, 10.88)	9.33 (6.19, 12.79)	0.040	7.55 (4.98, 11.87)	8.10(5.77, 14.13)	0.382	0.220
PCT [μg/L, M (QL, QU)]	0.05 (0.05, 0.24)	0.41 (0.05, 1.90)	<0.001	0.05 (0.05, 0.15)	0.21 (0.06, 0.81)	0.004	0.447
IG% [×109/L, M (QL, QU)]	0.40 (0.30, 0.60)	0.70 (0.40, 1.16)	<0.001	0.40 (0.30, 0.60)	0.70 (0.50, 1.30)	<0.001	0.812
APACHE II [M (QL, QU)]	3.00 (2.00, 5.00)	13.50 (9.75, 18.25)	<0.001	3.00 (2.00, 5.00)	13.00 (11.00, 20.00)	<0.001	0.893
SOFA [M (QL, QU)]	0.00(0.00, 1.00)	3.00 (2.00, 5.00)	<0.001	0.00 (0.00, 1.00)	3.00 (2.00, 5.00)	<0.001	0.276
Pathogenesis [n (%)]			0.437			0.437	0.279
Biliary origin [n (%)]	52.00 (38.20)	25.00 (27.80)		12.00 (24.50)	11.00 (40.70)		
Lipogenic [n (%)]	38.00 (27.90)	33.00 (36.70)		24.00 (49.00)	9.00 (33.30)		
Alcoholic [n (%)]	26.00 (19.10)	15.00 (16.70)		7.00 (14.30)	3.00 (11.10)		
Other [n (%)]	20.00 (14.70)	17.00 (18.90)		6.00 (12.20)	4.00 (14.80)		

*P*-value^1^ represents the *p*-value for the training cohort, *P*-value^2^ represents the *p*-value for the validation cohort, and *P*-value^3^ represents the *p*-value for the comparison of clinical characteristics between the training and validation cohorts.

Inflammatory markers and severity scores also showed significant differences. The MS-SAP group had significantly higher nCD64 index, CRP, PCT, IG%, APACHE II, and SOFA scores compared to the MAP group (*P* < 0.001 in both cohorts). White blood cell count (WBC) and neutrophil percentage (N%) showed marginal significance in the training cohort (*P* = 0.088 and *P* = 0.040, respectively), but these differences were not statistically significant in the validation cohort (*P* > 0.05).

Additionally, there were no significant differences in clinical characteristics between the training and validation cohorts (*P* > 0.05 in both cohorts).

### 3.2 Logistic regression, correlation, and ROC analysis

**Figure 3:**
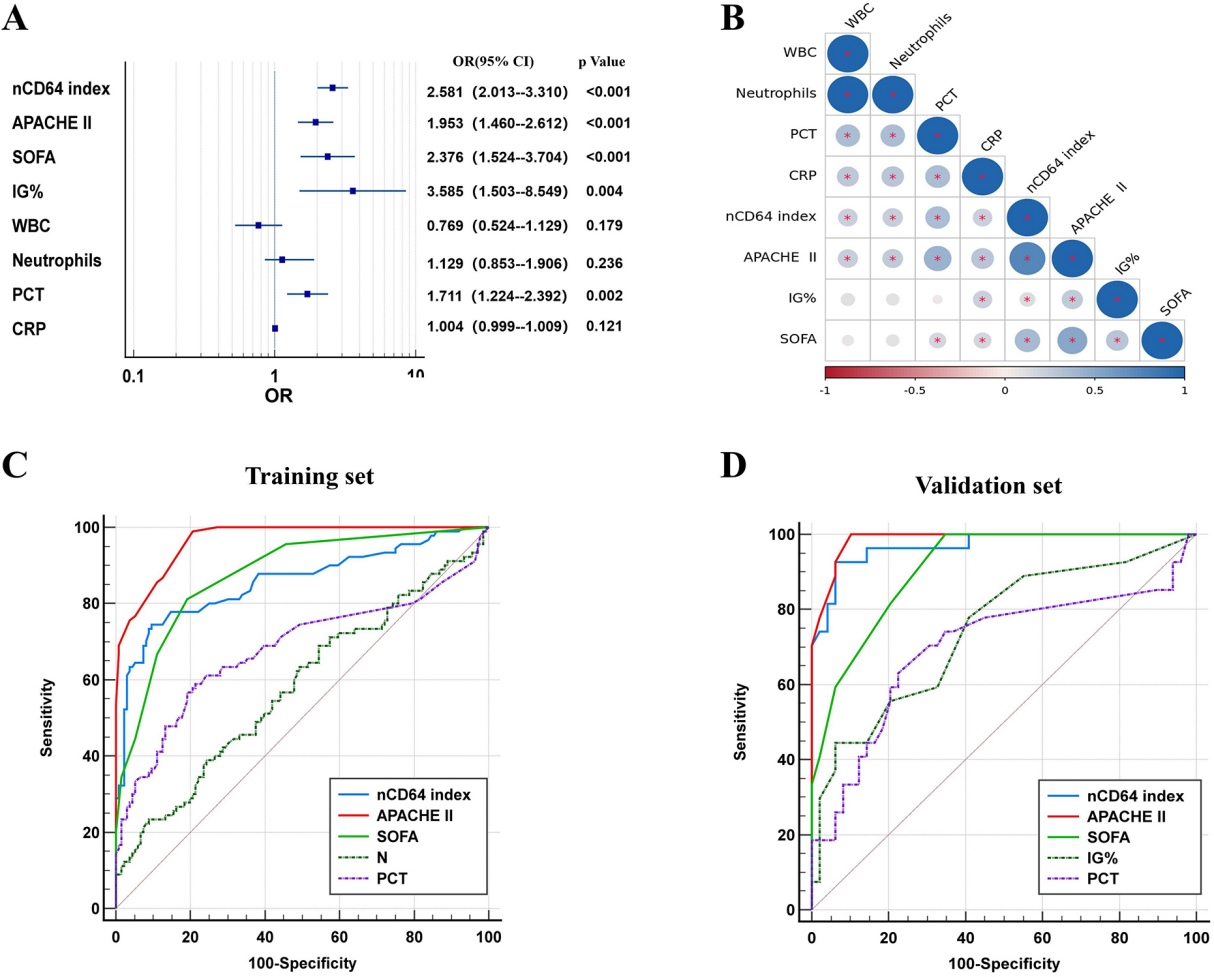
Logistic regression, correlation, and ROC analysis. **(A)** Forest map based on logstic; **(B)** Correlation coefficient chart; **(C)** ROC curve analysis of each index in the training cohort; **(D)** ROC curve analysis of each index in the validation cohort.

Logistic regression analysis identified elevated nCD64 index, PCT, APACHE II, SOFA scores, and IG% as independent risk factors for the progression to severe disease (**Figure 3A**). Notably, a strong positive correlation was observed between nCD64 index and APACHE II scores (**Figure 3B**, **Supplementary Table 1**), reinforcing the role of nCD64 in reflecting disease severity.

ROC curve analysis was conducted to evaluate the diagnostic performance of nCD64 index, PCT, APACHE II, SOFA scores, and IG%. The nCD64 index displayed the strongest diagnostic performance among the individual indicators. Although a combined indicator of nCD64 index, PCT, and IG% demonstrated excellent diagnostic accuracy, it did not significantly outperform the nCD64 index alone. The diagnostic efficacy of the nCD64 index was notably higher than that of IG% and PCT, and it was comparable to the APACHE II score. While the combined indicator showed superior diagnostic performance over IG% or PCT alone, it did not significantly differ from the nCD64 index when used independently (*P* > 0.05) (**Figures 3C, D**, **Table 2**). The cut-off value for the nCD64 index was determined to be 1.45, as presented in **Supplementary Tables 2** and **3**.

**Table 2:** Comparison of diagnostic efficiency of each index.

Comparing ROC curves	Study set	z statistic	Significance level
Comparing nCD64 index with APACHE-II	Training set	1.154	*P* =0.248
	Validation set	0.892	*P* = 0.373
Comparing nCD64 index with SOFA	Training set	2.533	*P* = 0.011
	Validation set	1.665	*P* = 0.100
Comparing nCD64 index with IG%	Training set	6.051	*P* < 0.001
	Validation set	3.901	*P* < 0.001
Comparing nCD64 index with PCT	Training set	6.455	*P* < 0.001
	Validation set	3.650	*P* < 0.001
Comparing nCD64 index with CRP	Training set	7.539	*P* < 0.001
	Validation set	4.093	*P* < 0.001
Comparing nCD64 index with nCD64 index + IG% + PCT	Training set	1.906	*P* = 0.057
	Validation set	0.560	*P* = 0.575
Comparing APACHE_II with nCD64 index + IG% + PCT	Training set	0.098	*P* = 0.922
	Validation set	0.765	*P* = 0.444
Comparing SOFA with nCD64 index + IG% + PCT	Training set	3.404	*P* < 0.001
	Validation set	1.827	*P* = 0.068
Comparing IG% with nCD64 index + IG% + PCT	Training set	7.097	*P* < 0.001
	Validation set	3.834	*P* < 0.001
Comparing PCT with nCD64 index + IG% + PCT	Training set	7.014	*P* < 0.001
	Validation set	3.792	*P* < 0.001

### 3.3 High-risk and low-risk group comparisons

**Figure 4:**
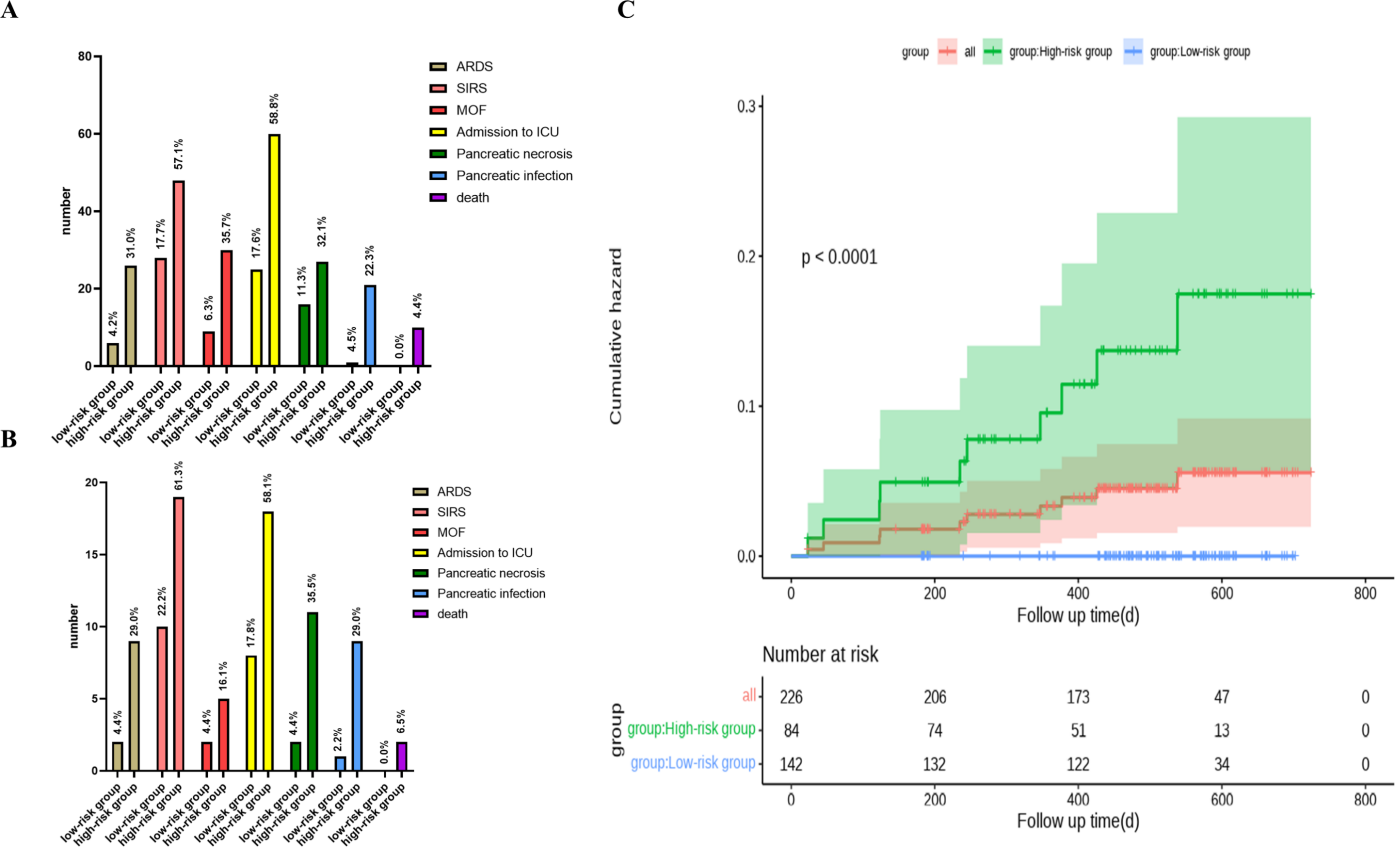
Clinical characteristics and cumulative hazard analysis of high-risk and low-risk patient groups. **(A)** Clinical features of all, high-risk, and low-risk groups. **(B)** Clinical features of high-risk vs. low-risk groups. **(C)** Kaplan-Meier plot depicting cumulative hazard for all, high-risk, and low-risk groups, with corresponding number of patients at risk over time shown below each group.

First, we conducted relevant analyses and determined the cutoff value for the nCD64 index (1.45) in the training cohort. We further confirmed these findings in the validation cohort. Therefore, we classified patients into high-risk (nCD64 index > 1.45) and low-risk (nCD64 index ≤ 1.45) groups using a cutoff value of 1.45. In the training cohort, patients in the high-risk group exhibited significantly higher rates of complications, including acute respiratory distress syndrome (ARDS), systemic inflammatory response syndrome (SIRS), multiple organ failure (MOF), pancreatic necrosis, pancreatic infection, ICU admission, and mortality, compared to those in the low-risk group. Specifically, 9 patients (29.0%) in the high-risk group developed ARDS, while only 2 patients (4.4%) in the low-risk group experienced ARDS, demonstrating a significant difference (*P* = 0.004). Similarly, 19 patients (61.3%) in the high-risk group developed SIRS, compared to 10 patients (22.2%) in the low-risk group (*P* = 0.001). The incidence of MOF was higher in the high-risk group (5 patients, 16.1%) compared to the low-risk group (2 patients, 4.4%), although this difference did not reach statistical significance (*P* = 0.093) (**Figure 4**).

ICU admission rates were also significantly higher in the high-risk group (58.1%) compared to the low-risk group (17.8%) (*P* < 0.001). Similarly, the rates of pancreatic necrosis and infection were much higher in the high-risk group, with pancreatic necrosis occurring in 35.5% of high-risk patients compared to only 4.4% in the low-risk group (*P* = 0.001), and pancreatic infection occurring in 29.0% of high-risk patients compared to 2.2% in the low-risk group (*P* = 0.001) (**Figure 4**).

Most importantly, over 65% of patients with an nCD64 index ≤ 1.45 were correctly classified as low-risk for mortality, highlighting the effectiveness of this stratification method in identifying patients with a lower probability of death. This finding was further confirmed in the validation cohort (**Figure 4**).

Kaplan-Meier survival curve analysis revealed a significant difference in survival outcomes between the high-risk and low-risk groups (*P* < 0.001). The survival rate was significantly lower in the high-risk group, further underscoring the utility of the nCD64 index as a prognostic marker for severe outcomes in acute pancreatitis (*P* < 0.001). Notably, the mortality rate in the high-risk group was 6.5%, while no deaths were observed in the low-risk group (*P* = 0.056) (**Figure 4**).

These findings further emphasize the prognostic value of the nCD64 index in stratifying patients by risk and guiding clinical management strategies.

## 4 Discussion

In this study, we observed notable differences in clinical characteristics, inflammatory markers, and severity scores between patients with MAP and those with MS-SAP, with the neutrophil CD64 index (nCD64) standing out as a particularly rapid, convenient, and reliable marker for assessing disease severity and prognosis. Through a thorough analysis of 302 acute pancreatitis (AP) patients, the nCD64 index was demonstrated to be a simple and effective biomarker that not only accurately reflects disease severity but also enables the early identification of high-risk patients, specifically those at increased risk of mortality.

### 4.1 Comparison with traditional scoring systems

Our study showed no significant differences in baseline characteristics such as age, gender, or etiology between the MAP and MS-SAP groups, suggesting that demographic factors and underlying causes of pancreatitis may not significantly influence disease severity (22, 23). However, the significantly longer hospital stays in the MS-SAP group highlighted the increased burden of care required for these patients, consistent with their more severe clinical manifestations. Inflammatory markers, especially the nCD64 index, CRP, and PCT, were markedly elevated in the MS-SAP group, indicating a higher inflammatory response. Among these, the nCD64 index, which reflects neutrophil activation, aligning with previous studies that have highlighted its sensitivity in detecting systemic inflammation and infection (16).

Traditional scoring systems such as APACHE II and SOFA are time-consuming and require extensive clinical data (24–26). In contrast, the nCD64 index showed a strong positive correlation with the APACHE II score, a widely used tool for assessing disease severity, suggesting that nCD64 could offer a simpler and more accessible alternative for rapid risk stratification (27–30). Unlike these complex systems, the nCD64 index can be quickly measured through routine blood tests, delivering fast and actionable clinical insights.

### 4.2 Cutoff Value of nCD64 and Identification of High-Risk Patients

The study identified an optimal nCD64 cutoff value of 1.45, above which patients were significantly more likely to experience life-threatening complications, such as systemic inflammatory response syndrome (SIRS), acute respiratory distress syndrome (ARDS), and multiple organ failure (MOF). These patients also faced a notably higher risk of mortality. This finding aligns with previous studies, confirming that nCD64 is a sensitive marker for systemic inflammation and infection. By using this threshold, clinicians can effectively distinguish high-risk patients who require intensive care and timely intervention to potentially improve outcomes.

Further analysis revealed that while a composite marker (nCD64 index + PCT + IG%) improved diagnostic efficacy compared to individual markers, it did not significantly outperform the nCD64 index alone. This underscores the practicality of using the nCD64 index as a standalone marker. Moreover, we identified a cut-off value of 1.45 for the nCD64 index, which effectively differentiated between high- and low-risk groups. Kaplan-Meier survival analysis showed that patients in the high-risk group (nCD64 > 1.45) had significantly poorer survival outcomes, emphasizing the prognostic value of the nCD64 index for predicting mortality.

### 4.3 Application of nCD64 in Risk Stratification

Kaplan-Meier survival analysis further underscored the clinical significance of the nCD64 index. Patients in the high-risk group (nCD64 > 1.45) had significantly poorer survival outcomes compared to those in the low-risk group (p<0.001). This demonstrates that the nCD64 index can not only aid in early diagnosis but also serve as a valuable prognostic tool. Early identification of high-risk patients allows for prompt intervention, including close monitoring and early admission to the ICU, thereby improving patient survival (31, 32).

Although this study demonstrated the validity of nCD64 in a Chinese cohort, further research is needed to confirm its generalizability across different populations and healthcare systems. Future studies should also explore combining nCD64 with other biomarkers to refine diagnostic and prognostic models.

The limitations can be written in the following way in the article:This study has certain limitations in data sources and patient population. The data were derived from only two medical centers in Hunan Province (Hunan Provincial People’s Hospital and Changsha Central Hospital). Although these centers represent diversity in patient demographics and clinical expertise, the geographic and healthcare environment coverage remains limited compared to a true multicenter study. In future research, we will actively collaborate with institutions across diverse regions and healthcare settings to validate the generalizability and applicability of our findings in broader patient populations.

In conclusion, the nCD64 index is a powerful and efficient tool for identifying high-risk patients in acute pancreatitis. Its simplicity and strong correlation with established severity scores make it a valuable addition to traditional scoring systems. Incorporating the nCD64 index into standard clinical protocols could enhance early identification of high-risk patients, optimize resource allocation, and ultimately improve patient outcomes.

## Data Availability

The original contributions presented in the study are included in the article/**Supplementary Material**. Further inquiries can be directed to the corresponding author/s.
