# Supplementary material for: Neutrophil CD64 index: a novel biomarker for risk stratification in acute pancreatitis
Source: Front Immunol. 2025 Apr 16;16:1526122. doi: 10.3389/fimmu.2025.1526122 (PMC12040616; doi:10.3389/fimmu.2025.1526122)
Supplement: Supplementary file 1 [file Table1.docx]

**Supplementary Table 1. Correlation analysis**

| **Correlation** | | | | | | | | | |
| --- | --- | --- | --- | --- | --- | --- | --- | --- | --- |
|  | | nCD64 index | APACHE II | SOFA | IG% | WBC | N | PCT | CRP |
| nCD64 index | Pearson correlation | 1 | .766^**^ | .403^**^ | .206^**^ | .178^**^ | .201^**^ | .331^**^ | .213^**^ |
|  | Sig. (Double tail) |  | .000 | .000 | .000 | .002 | .000 | .000 | .000 |
| APACHE II | Pearson correlation | .766^**^ | 1 | .510^**^ | .273^**^ | .176^**^ | .195^**^ | .403^**^ | .281^**^ |
|  | Sig. (Double tail) | .000 |  | .000 | .000 | .002 | .001 | .000 | .000 |
| SOFA | Pearson correlation | .403^**^ | .510^**^ | 1 | .263^**^ | .085 | .109 | .204^**^ | .171^**^ |
|  | Sig. (Double tail) | .000 | .000 |  | .000 | .139 | .058 | .000 | .003 |
| IG% | Pearson correlation | .206^**^ | .273^**^ | .263^**^ | 1 | .089 | .076 | .063 | .216^**^ |
|  | Sig. (Double tail) | .000 | .000 | .000 |  | .121 | .185 | .273 | .000 |
| WBC | Pearson correlation | .178^**^ | .176^**^ | .085 | .089 | 1 | .981^**^ | .312^**^ | .294^**^ |
|  | Sig. (Double tail) | .002 | .002 | .139 | .121 |  | .000 | .000 | .000 |
| N | Pearson correlation | .201^**^ | .195^**^ | .109 | .076 | .981^**^ | 1 | .312^**^ | .302^**^ |
|  | Sig. (Double tail) | .000 | .001 | .058 | .185 | .000 |  | .000 | .000 |
| PCT | Pearson correlation | .331^**^ | .403^**^ | .204^**^ | .063 | .312^**^ | .312^**^ | 1 | .333^**^ |
|  | Sig. (Double tail) | .000 | .000 | .000 | .273 | .000 | .000 |  | .000 |
| CRP | Pearson correlation | .213^**^ | .281^**^ | .171^**^ | .216^**^ | .294^**^ | .302^**^ | .333^**^ | 1 |
|  | Sig. (Double tail) | .000 | .000 | .003 | .000 | .000 | .000 | .000 |  |
| **. At level 0.01 (two-tailed), the correlation was significant. | | | | | | | | | |
