# Supplementary material for: Neutrophil CD64 index: a novel biomarker for risk stratification in acute pancreatitis
Source: Front Immunol. 2025 Apr 16;16:1526122. doi: 10.3389/fimmu.2025.1526122 (PMC12040616; doi:10.3389/fimmu.2025.1526122)
Supplement: Supplementary file 2 [file Table2.docx]

| index | AUC | CUT-OFF | Sensitivity (%) | Sensitivity (95% CI) | Specificity (%) | Specificity (95% CI) | +LR | +LR (95% CI) | -LR | -LR (95% CI) |
| --- | --- | --- | --- | --- | --- | --- | --- | --- | --- | --- |
| nCD64 index | 0.948 | 1.45 | 86.67 | 77.9 - 92.9 | 95.59 | 90.6 - 98.4 | 19.64 | 8.95 - 43.13 | 0.14 | 0.082 - 0.24 |
| APACHEⅡ | 0.966 | 5.00 | 98.89 | 94.0 - 100.0 | 79.41 | 71.6 - 85.9 | 4.80 | 3.45 - 6.69 | 0.014 | 0.0020 - 0.098 |
| SOFA | 0.877 | 1.00 | 81.11 | 71.5 - 88.6 | 80.88 | 73.3 - 87.1 | 4.24 | 2.96 - 6.08 | 0.23 | 0.15 - 0.36 |
| IG% | 0.724 | 0.60 | 52.22 | 41.4 - 62.9 | 80.15 | 72.4 - 86.5 | 2.63 | 1.78 - 3.89 | 0.60 | 0.47 - 0.75 |
| PCT | 0.681 | 0.20 | 58.89 | 48.0 - 69.2 | 78.68 | 70.8 - 85.2 | 2.76 | 1.92 - 3.98 | 0.52 | 0.40 - 0.68 |
| CRP | 0.696 | 27.25 | 77.78 | 67.2 - 86.3 | 55.37 | 46.1 - 64.4 | 1.74 | 1.38 - 2.19 | 0.40 | 0.26 - 0.62 |
| nCD64 index  +IG%+PCT | 0.965 | 0.56 | 87.78 | 79.2 - 93.7 | 98.53 | 94.8 - 99.8 | 59.69 | 15.05 - 236.75 | 0.12 | 0.071 - 0.22 |

**Supplementary Table 2. Analysis of diagnostic value of corresponding indexes of acute pancreatitis severity in training cohort**
