# Supplementary material for: Neutrophil CD64 index: a novel biomarker for risk stratification in acute pancreatitis
Source: Front Immunol. 2025 Apr 16;16:1526122. doi: 10.3389/fimmu.2025.1526122 (PMC12040616; doi:10.3389/fimmu.2025.1526122)
Supplement: Supplementary file 3 [file Table3.docx]

**Supplementary Table 3. Analysis of the diagnostic value of the corresponding indicators of the severity of acute pancreatitis in the validated cohort**

| index | AUC | CUT-OFF | Sensitivity (%) | Sensitivity (95% CI) | Specificity (%) | Specificity (95% CI) | +LR | +LR (95% CI) | -LR | -LR (95% CI) |
| --- | --- | --- | --- | --- | --- | --- | --- | --- | --- | --- |
| nCD64 index | 0.969 | 1.45 | 92.59 | 75.7 - 99.1 | 93.88 | 83.1 - 98.7 | 15.12 | 5.03 - 45.51 | 0.079 | 0.021 - 0.30 |
| APACHEⅡ | 0.986 | 5.00 | 92.59 | 75.7 - 99.1 | 93.88 | 83.1 - 98.7 | 15.12 | 5.03 - 45.51 | 0.079 | 0.021 - 0.30 |
| SOFA | 0.911 | 1.00 | 81.48 | 61.9 - 93.7 | 79.59 | 65.7 - 89.8 | 3.99 | 2.23 - 7.14 | 0.23 | 0.10 - 0.52 |
| IG% | 0.745 | 0.60 | 44.44 | 25.5 - 64.7 | 93.88 | 83.1 - 98.7 | 7.26 | 2.24 - 23.50 | 0.59 | 0.42 - 0.84 |
| PCT | 0.700 | 0.20 | 62.96 | 42.4 - 80.6 | 77.55 | 63.4 - 88.2 | 2.80 | 1.55 - 5.09 | 0.48 | 0.29 - 0.80 |
| CRP | 0.753 | 19.90 | 88.89 | 70.8 - 97.6 | 59.18 | 44.2 - 73.0 | 2.18 | 1.52 - 3.13 | 0.19 | 0.063 - 0.56 |
| nCD64 index  +IG%+PCT | 0.973 | 0.56 | 96.30 | 81.0 - 99.9 | 91.84 | 80.4 - 97.7 | 11.80 | 4.60 - 30.26 | 0.040 | 0.0059 - 0.28 |
