# Supplementary material for: Neutrophil CD64 index: a novel biomarker for risk stratification in acute pancreatitis
Source: Front Immunol. 2025 Apr 16;16:1526122. doi: 10.3389/fimmu.2025.1526122 (PMC12040616; doi:10.3389/fimmu.2025.1526122)
Supplement: Supplementary file 4 [file DataSheet1.pdf]

frontiers

The International Society for the Advancement of Cytometry (ISAC) has highlighted the importance of including comprehensive methodological information to ensure data reproducibility and reliability. In line with this, Frontiers in Immunology now requires authors to submit a checklist for manuscripts that involve flow or mass cytometry. This checklist helps standardize the reporting process, improving the quality and transparency of published data. By doing so, we support scientific progress, making it easier for other researchers to replicate and validate experiments.

This form should be submitted with any manuscripts using flow or mass cytometry.

Sample/specimen/material description

- [x] Total blood
- [ ] PBMCs
- [ ] Organ digests

Other____

Did the samples suffer any treatment before or after incubation with the antibodies?

- [ ] Drug ______________________________________
- [ ] Cell permeabilization ________________________
- [ ] Dye _______________________________________
- [ ] Propidium iodine
- [x] Not applicable

Other ____

Instrument and antibodies

Name of the Cytometer Instrument(Myriad BriCyteE6, Mindray Medical, Shenzhen, China)

Antibodies and targets	Fluorochrome/ Metal	Catalog number/Company
e.g. anti-CD4	FITC	Cat. XXX/ XXX ltd.
PerCP Anti-Human CD14	FC	399512/BioLegend

PerCP Anti-Human CD45	FC	325632/BioLegend
PerCP Anti-Human CD64	FC	304026/BioLegend

Data analyses

1. Name of the software____FlowJo V10______________
2. Reference gating strategy in the manuscript or supplementary material

Gating strategy in (eg Figure X) ______Figure 2________________
